# Transdisciplinary solutions to methodological and ethical problems in performing arts psychology

**DOI:** 10.3389/fpsyg.2023.1330479

**Published:** 2024-01-16

**Authors:** Pil Hansen

**Affiliations:** School of Creative and Performing Arts, University of Calgary, Calgary, AB, Canada

**Keywords:** performing arts, psychology, research methods, transdisciplinary, research ethics, diversity and inclusion, relational ethics, practice-based research

## Abstract

In the first part of this article, I argue that transdisciplinary research can help address both the WEIRD problem of homogenous samples and the categorical problem of overgeneralized practice conceptions in performing arts psychology. Like other areas of performance science, performing arts psychology engages with practices and practice-based knowledge that are studied differently by subject-specific disciplines. I propose a transdisciplinary research model that facilitates greater overlap and transfer between the scientific, subject-specific, and practice-based forms of research and knowledge that surround a practice. The potential benefits of such a model are more inclusive samples, diversified methods, grounded research questions, and widely applicable results. The problems mentioned above are also ethical. Psychological definitions of performance that derive from overgeneralized conceptions and overreliance on homogenous samples are transferred to diverse peoples, practices, and contexts as general knowledge. This fails to apply principles of equity and relational ethics, which in turn reveals some limitations of established ethics procedures. In the second section of this article, I therefore revisit my argument for transdisciplinary research, now with a focus on the triad of research ethics that is brought into a transdisciplinary project through the different priorities of scientific, subject-specific, and practice-based research domains; namely, procedural ethics, relational ethics, and principles of equity. Transdisciplinary researchers are not only negotiating across methodological paradigms that determine research validity, they are also negotiating across ethical values. Combining the two sections of the article, I argue that the challenge of negotiation can be flipped into a solution to the WEIRD and practice conception problems in performing arts psychology. I argue that whereas critical calls for radical departures were needed to identify these problems, solutions are available in bridges between different ethical and methodological approaches.

## 1 Introduction

Performing arts psychology inherited both the WEIRD problem of homogenous samples and the problem of overgeneralized practice conceptions from the discipline of psychology. However, the field of performing arts psychology can more readily aim to address these problems through transdisciplinary methodology and ethics due to its proximity to performing arts disciplines. As I argue in this perspective article, each discipline brings strengths and limitations to the problem, but together they enable both increased diversification and improved practice specificity.

## 2 Transdisciplinary solutions to methodological problems

### 2.1 The methodological problem of homogenized samples

In their recent book, *Psychology's WEIRD Problem*, de Oliveira and Baggs (2023) deliver a thorough discussion of the problems behind homogenous samples in psychology. The WEIRD acronym was coined by Henrich and colleagues in 2010 to consolidate critique of overreliance on study participants from “Western, Educated, Industrialized, Rich, Democratic” societies (Henrich et al., 2010)^1^. The critique has reached popular awareness as the problem of conveniently recruiting study participants among psychology students in the Western world. The consequence of this tendency is, arguably, that universalized cognitive functions neither reflect people as they are most, nor peoples in their diversity, but rather the statistical commonalities of a comparatively unique and small subgroup (de Oliveira and Baggs, 2023, p. 7).

de Oliveira and Baggs argue that the established critique was cast too narrowly because the lack of diversity in samples is encouraged by systemic norms in psychology, such as the metrics for assessing researchers' impact and the standardized research methods used. To recruit diverse samples inclusively, and thus base theories about human psychology on a less narrow demographic, relationships would have to be built with communities outside the university and multi-site or multi-partner research would be required (de Oliveira and Baggs, 2023, p. 3–6). Such studies are time- and resource-demanding, and complex variables render them less likely to produce statistically valid results. In other words, they do not yield grants, proven hypotheses, or publications with the efficiency that builds academic careers. de Oliveira and Baggs also critique the historical turn in psychology to rely on experiments as stand-alone methods. If hypotheses are to include more diverse samples, de Oliveira and Baggs argue, they should not be formed within WEIRD research teams and academies alone. Instead, hypotheses for experimental testing would need to be grounded meaningfully in sites and communities, through methods such as participatory research that include the knowledge and interests of diverse communities (de Oliveira and Baggs, 2023, p. 55)^2^.

A more radical turn to participatory research methodology is taking place in critical ethnography and social psychology (e.g., Cockburn and Cundill, 2018; Allen et al., 2022; Kessi et al., 2022). To ask such a turn of experimental psychology, however, would involve a risk of losing access to insights that are not evident in inter-personal exchanges or register consciously in a community context. I am thinking of implicit, cognitive, and perceptual processes. That is perhaps why Oliveira and Braggs instead argue for methodological diversity that provides complementary access to situated knowledge (de Oliveira and Baggs, 2023, p. 37, 55).

### 2.2 The methodological problem of overgeneralized conceptions

The problem of overgeneralization in performing arts psychology has been apparent in my editorial work over the past decade. In dance and theater psychology, for example, I find that authors often operate with a generalized concept of dance, acting, or human action, despite drawing on a narrow dance or acting form for interventions (e.g., Sumanapala and Cross, 2017. See Discussion in Hansen and Bläsing, 2017; Jola and Hansen, 2021). This anecdotal observation was recently corroborated empirically in the findings of a bibliometric review of cognitive dance studies (Warburton, 2023). To explain the problem, I briefly elaborate on examples from dance. Technique and inter-dancer relations in dance forms taught in Western institutions, such as codified ballet, open contact improvisation, or task-based contemporary dance, are fundamentally dissimilar and, therefore, place different physical and cognitive demands on dancers. For example, some of these forms require precise memorization and recall, whereas others require continuous attention shifting and response in the present (see Hansen, 2022). When considering dance forms on a global scale, the differences become more significant. Concluding something about dance in general based on a ballet intervention alone is therefore to overreach. Without awareness of the demands that a particular dance form may place on dancers, hypotheses about the psychological dimensions of the practice may also come up against validity limitations. In application, this problem has, for example, led to dance interventions for the social inclusion of older adults with dementia in which a young dancer demonstrates contemporary dance movement that is mirrored by older adults lined up on rows (e.g., Skinner et al., 2018; see also Hansen et al., 2021). More situated attention to socially engaging and meaningful dance forms can lead to interactive movement drawn from dances that were popular in the older adults' youth and culture (Lima and Vieira, 2007). These examples are related to the WEIRD problem described in the previous section, as the overgeneralized concepts of dance are caused by a distance from the specific artforms and the diverse socio-cultural contexts in which they are practiced.

### 2.3 Characteristics of performing arts psychology that invite transdisciplinarity

Transdisciplinary research approaches (TDA) aim to understand and solve complex problems by transgressing the boundaries of individual disciplines and approaching the problems holistically. Instead of primarily transferring a method or theory into an otherwise intact discipline (as in multi-disciplinary research) or pursuing singular points of integration (as in interdisciplinary research), TDA aims to co-produce knowledge, combining methods, and theory from multiple disciplines, and working closely with partners in society (Jahn et al., 2012; Rezaei and Seyedpour, 2022).

Performing arts psychology, and performance psychology more broadly, examine psychological aspects of various practices that are enacted in society. When applied, the psychological insights produced often help advance the practices or transfer them to new socio-cultural contexts. When we approach “society” in transdisciplinary research models in ways that include practice-based knowledge and socio-culturally situated knowledge, I have repeatedly found that transdisciplinary collaboration can help reduce the risks of overgeneralization and/or homogenous sampling (e.g., Stevens, 2005; Shaughnessy, 2012; Hansen et al., 2020, 2021). Whereas, participatory methods in social psychology can help researchers engage meaningfully with communities of practice (e.g., Allen et al., 2022), subject-specific research disciplines in the performing arts have insights and methods to offer that are more closely associated with the performance practice, and thus also important to help solve the mentioned problems.

In performing arts research, fields such as dance studies, musicology, theater studies, and performance studies research a shared subject, the artform. They use a broad range of methods selectively, many of which are drawn from other disciplines and adapted. The priority of these fields is not methodological consistency, replicability, or generalizability, but rather the production of varied epistemological perspectives on the subject matter. It can be likened to holding up a multifaceted prism to the artform and examining how interpretations change depending on the chosen angle. Subject-specificity and context are important for the validity of this work, and thus the research yields deep and detail-oriented analyses of practices that are situated socially, culturally, geographically, and historically. While full methodological transparency is rare, explicit use of theoretical frameworks is more common with an interest in how a new theory may critically change or diversify what a practice possibly can do and how we may interpret it (Candelero and Henley, 2023). While such theory often is drawn from Western philosophy, attention to theory from the Global South and Indigenous ways of knowing has grown considerably over the past two decades. By collaborating with subject-specific fields, performing arts psychology can gain situated, contextualized, and diversified insight into the various practices studied and peoples involved^3^.

Over the past two decades, these subject-specific fields have, furthermore, developed relevant practice-based research approaches (Nelson, 2013; Barton, 2017; Midgelow et al., 2019)^4^. Those methods often produce emergent, performative knowledge over iterative cycles of creating, observing, and reflecting. The work involves creating and bringing something into the world through embodied engagement with a series of inquiries and practices, while remaining observant and reflexive about the contextual resources drawn on, the choices made, strategies used, performances produced, and any other aspects that pertain to the inquiry. Creative research methods are combined with methods of observation and analysis, drawn selectively from empirical and theoretical disciplines. By including such practice-based research methods in TDR, practitioners' knowledge is brought into the study design, the development of interventions, and the discussion of possible steps toward application of results.

### 2.4 Multi-methodological and transdisciplinary model as change driver

In 2009, Bruce Barton and I co-developed a model titled Research-Based Practice (RBP), intended for research that crosses cognitive psychology and performing arts creation practice. The model involves multiple interdisciplinary (or discipline-specific) teams working on a shared set of research problems, empirical/artistic practices, and samples/sites. However, they pursue their research in separate spaces, each of which is designed adhering to the methodological standards of the primary discipline within the team. The idea was that results, new questions, and data sets from these separate spaces are transferred to a 3rd space, in which all collaborators explore analytical and creative possibilities without reference to a specific methodological framework. Abductive^5^ leaps made in the 3rd space can then be brought back into the methodologically defined spaces for further development or validation. In 2017, I updating this model based on experiences gained over a decade of application. Updates include the role practice-based research can have in (1) examining mediating processes (that is, the factors that cause measured effects), and (2) developing strategies for application of findings to practice in society (Hansen, 2017).

Rather than waiting for institutional and material conditions to fully undergo the changes that de Oliviera and Baggs call for, the RBP model acknowledges (1) that different methodologies can examine different aspects of a phenomenon, including aspects that otherwise are hidden by the WEIRD paradigm; and (2) that combining methodologies therefore can be a useful driver of diversification. Instead of hoping to integrate different disciplines under a single epistemological framework, our model is recognized for enabling collaborators to produce multiple kinds of knowledge through productive inter-disciplinary discovery and collaboration (e.g., Ciesielski, 2023; Murphy, 2023). When the different results and the interdisciplinary exchange are brought together in response to the original and shared research questions and practices studied, then we arrive at transdisciplinary knowledge. However, individual parts, enriched by the transdisciplinary process, remain recognizable as valid knowledge and publishable or applicable within singular disciplines and practice-fields. This allows research to become funded and have impact within the constraints de Oliviera and Braggs critique, while also gaining capacity to address the WEIRD problem and the problem of overgeneralization.

### 2.5 Grounded, diversified, and transparently negotiated methods

Transdisciplinary frameworks like the RBP model may begin to address some of the systemic factors that produce WEIRD bias from within. When such frameworks are used in performing arts psychology to bring subject-specific and practice-based forms of knowledge and research into collaboration with scientific inquiry, then grounding in specific practices and contexts of practice become stronger (see Ciesielski, 2023; Murphy, 2023; Shaughnessy et al., 2023). This furthermore means that homogenized sample groups no longer can remain hidden under universalized findings, as the specific practices, practice-context, and its associated sample group will be more transparent.

It follows that conceptions of the practice therefore also will become both more accurate and recognizable to the sample group. With more grounded practice conceptions, research hypotheses gain construct validity and experimental interventions are more likely to yield the hypothesized effects. The community context, which practices and samples are drawn from, may also be more likely to apply findings when conceptions are more recognizable, and/or representatives have been involved as community partners and participatory or practice-based researchers. This factor can further increase the likelihood of impact and may therefore offset the risks and additional work involved in transdisciplinary research.

In my opinion, transparency and specificity are also preconditions for diversity. They reveal the limitations of too homogenous samples in ways that place material pressure on researchers and funders to expand the transferability of their findings by working across multiple sites and contexts instead of relying on universalism. More importantly, they require researchers to consider findings of WEIRD studies equally specific to a group and thus become more sensitive to the variables of other groups.

Another useful feature is that frameworks like PBR render points of methodological negotiation clearer (see Hansen and Bläsing, 2017; Ciesielski, 2023). Each discipline brings a set of priorities that must be met for validity within their research space, whereas standards that are less important can be released in support of another discipline's priorities. Acknowledging and articulating methodological differences with shared investment is, in my experience, a precondition for transcending disciplines successfully. Doing so within a model that produces multiple kinds of related knowledge for both discipline-specific and transdisciplinary dissemination helps address the WEIRD problem despite systemic constraints.

## 3 Transdisciplinary solutions to ethical problems

### 3.1 The ethical problems of homogenized samples and overgeneralized conceptions

The WEIRD problem is also ethical. When a fields' understanding of psychological processes is based on a too homogenous sample, then drawing universal conclusions and applying them globally is not only a threat to validity. In the best case, some population groups become invisible to the research field because they are excluded from the universalized knowledge on which theory is based. In the worst case, such excluded populations may also be misunderstood or mistreated when universalized knowledge is applied to practices, education, funding, policies, institutional structures, and more. Ethically, this means that principles of inclusion and equity are not met.

Such principles may be constitutional rights, embedded in the policies of research institutions and funding bodies, and/or prioritized by research associations and publishers. They can be difficult to consider for a research ethics review board as the review process, understandably, focuses on reducing risks of harm to the direct participants in the study. Risk of harm to groups that are external to the study sample, but implicated via generalization, is not (yet) considered in most procedural ethics reviews. This argument extends to the problem of overgeneralized conceptions in performing arts psychology, but here it is diverse practices that are excluded. Add the two problems up, and the barriers to equity and diversity stand clearer.

Although de Oliveira and Braggs do not frame their critique in terms of ethics and therefore miss the ethical dimensions I contour here, their advocacy for methodological diversity remains relevant. Returning to the transdisciplinary solution for achieving this diversity in performing arts psychology, disciplines that draw on either scientific, critical, or practice-based methodologies to research the performing arts have different ethical strengths and limitations. Combined, they may be better equipped to address the ethical problems named above.

### 3.2 The triad of ethics in transdisciplinary and practice-based research: procedural ethics, relational ethics, and equity principles

In performing arts psychology, study designs are informed by procedural standards for ethics from the earliest stage of planning. Relational ethics and equity are less commonly considered. This tendency is reflected in recent books on research methods in the dance sciences and in performance and music psychology that exclusively include ethics chapters on protocol and review procedures (Williamon et al., 2021; Welsh et al., 2023). As emphasized in these textbooks, ethics policies and standards for review have been adopted by research funders and universities in many countries. Such standards are typically framed around the principles identified in the 1970s American Belmont Report: (1) respect for persons, (2) beneficence, and (3) justice. In ethics protocols, these principles tend to translate to: (1) high standards for the respectful, informed, and voluntary consent of participants and protection of confidentiality; (2) ensuring that participants' risks are minimized and outweighed by direct and indirect benefits, (3) providing equitable access to participation benefits and distributing risks and burdens equitably, while avoiding harm to vulnerable groups. These procedures are meant to ensure researchers do not repeat historical practices that have caused harm (Pritchard, 2021).

In Canada, practice-based research that produces artistic works is exempt from ethics review. In much of Europe, the inclusion of artistic educations under university institutions is comparatively new. Under these circumstances, artistic researchers established a norm of engaging with artistic and practice-based inquiry first, and fitting it into institutional ethics procedures second. The procedures of ethics boards have been developed based on scientific standards, where large-scale, anonymous, and randomized studies with control groups are deemed more likely to manage risks and produce beneficial results. Ethics procedures furthermore require researchers to describe (and thus determine) what participants will experience up front. As documented by MacNeill and colleagues, these factors have led artistic researchers in the performing arts to view ethics review as a barrier to their more open research processes (MacNeill et al., 2021, p. 81; also see Rice et al., 2018).

Artistic, practice-based research is typically generative and carried out over iterative cycles. Although general plans can be described in the design phase, research activities will undergo change as the plans for later cycles are adapted in response to discoveries and creation from earlier ones (see Nelson, 2013; Hansen, 2017). Activities may depend on choices made among participants during the process, rather than the plans made by the lead artist-researcher up front. The negative experience of ethics review procedures described above therefore changes when artistic researchers and ethics reviewers are able to build bridges between procedural ethics and the form of ethics that is more developed in artistic research: namely relational ethics (MacNeill et al., 2021, p. 80). Here, consent is typically ongoing, it is renewed at each encounter with participants and collaborators, and may even be renewed multiple times over a single encounter when performance creation activities involve touch, the sharing of personal experience, or engagement with sensitive topics. Professional codes and practices around agency, decision making, pay, work hours, and much more also partake in relational ethics. Guidelines and frameworks for how a group of artists work together and produce a safe space are referred to when decisions have to be made on short notice (MacNeill et al., 2021, p. 85; Hansen, 2023).

Relational ethics in performing arts research share some characteristics with participatory research methods in social psychology, which may facilitate transdisciplinary collaboration. In both fields, participatory decision making can range from meaningful consultation and ongoing consent in studies led by researchers (e.g., Mirfin-Veitch et al., 2018; Cwik, 2021; Pavarini et al., 2021) through to co-design, co-leadership, and co-authorship of all research plans and outcomes (e.g., Hibberd, 2020; Allen et al., 2022). As indicated by the examples cited above, considerations of equity, including principles of diversity, inclusion, and accessibility (EDIA), can be determining for where a project's relational ethics are positioned on this range.

Returning to subject-specific performing arts research, this is particularly true in qualitative, applied studies in the performing arts that draw on the most recent critical theory paradigms in dance studies, musicology, theater studies, and performance studies (e.g., critical race theory, decolonization theory, critical gender theory, and critical disability theory; Cox et al., 2020; Laukkanen et al., 2022; Sadeghi-Yekta and Prendergast, 2022). These frameworks require awareness of systemic marginalization and implicit bias as well as commitment to deconstruct and counter such tendencies for ethical purposes or to affect transformative, social change. Such theoretical lenses are reflected in institutional changes that currently are emerging within Western universities and fundings bodies; changes which solicit both positive and negative awareness of equity principles. EDIA committees are established, EDIA priorities are adopted in strategic plans, and EDIA awards are launched. Political counter-reactions also emerge, causing legislation against EDIA work in some contexts where equity is not a constitutional right.

As Macleod et al. (2018, p. 9) note, positive EDIA awareness does not safeguard against harm. Despite best intentions to undo past harms by, for example, repositioning marginalized groups at the center, it is important to be mindful that EDIA-aware research can have unintended negative effects. For example, participants may not wish to be framed theoretically or defined by identity markers; norms that are deconstructed may be valued differently in the community; the inclusion of one group may exclude another; and research instruments tend to assume researcher-participant hierarchies that can hinder relational ethics. In my opinion, EDIA awareness must be translated into explicit and operational guidelines for ongoing bias screening and relational choice making to reduce risks of harm. Bolt and Vincs (2015, p. 1,305) raise a similar concern about practice-based research in the arts, when they argue that “research methodology and design are fundamental to the development of ethical research.”

Drawing on experiences from applied performing arts research (Indigenous, educational, and practice-based), Kirsten Sadeghi-Yekta and Monica Prendergast, among others, suggest a series of themes that are helpful when developing relational ethics guidelines and study designs. Caring for and being of service to the people and more-than-people who participate in the research are considered more important than research objectives that are external to the interests of participants. A continuous practice of listening to participants and adjusting plans accordingly is essential. Efforts are made to render listening inclusive, which means considering the many forms of expression and languages involved, verbal and non-verbal, embodied in practice or embedded in space. Adjusting plans often means noticing when to slow down or change path. Reciprocal reflection also reemerges across the literature, and a wealth of examples are shared of how culturally meaningful practices can become reflection formats (from storytelling circles through artistic feed-back models). Methods of self-reflection and ongoing bias screening are raised, and so are ideas for how to share research agency and ownership with participants (Cox et al., 2020; Hibberd, 2020; Timonen et al., 2021; Laukkanen et al., 2022; Ruby et al., 2022; Sadeghi-Yekta and Prendergast, 2022).

Guidelines for such relational ethics are not required by ethics review boards, nor directly deriving from critical theoretical paradigms. Indeed, adaptive research objectives and methods may be difficult for an ethics board to approve, and sharing agency can require a researcher to relax or let go of a pre-defined theoretical framework. That being said, the triadic spheres of ethics are not mutually exclusive. Relational ethics do live up to the procedural ethics principles of respect, beneficence, and justice. The decentering of research hierarchies and radical inclusivity involved in relational ethics are also closely aligned with equity principles.

### 3.3 Ethics procedures with relational and equity dimensions

A transdisciplinary approach to ethics in performing arts psychology may provide a solution to the ethical problems previously discussed. Particularly, insofar the strengths and limitations of ethics review procedures, relational ethics positions, and EDIA frameworks are fully considered, and complementary connections are established. Procedural ethics cannot safeguard against negative effects for population groups that are external to the study. Indeed, some of the methodological standards embedded in procedural ethics can inhibit solutions. However, the requirement to establish clear guidelines for consent, participation, and benefits could productively be expanded with guidelines for bias screening, inclusion, and ongoing consent in relational and participatory research. Critical theoretical frameworks and EDIA awareness reveal risks of harm to external groups, can promote greater diversity, and can also motivate attempts to not only avoid but also recognize and deconstruct harmful past practices. However, these frameworks require operational procedures and flexible, relational anchoring to safeguard against unintended harm. When EDIA awareness and relational ethics are combined, the research design is often more deeply grounded in the participants' reality and belief systems, drawing on multiple forms of participant-as-researcher collaboration. Again, establishing clear procedures and guidelines with these participants that can be referred to jointly as the work progresses is needed to ground the research meaningfully. It can also help ensure that epistemological and ontological assumptions of theoretical frameworks and methods do not overwrite population groups with WEIRD or overgeneralized ways of knowing.

## 4 Discussion: turning transdisciplinary negotiation into a solution

Instead of reiterating calls for a radical turn to a participatory research paradigm with an emphasis on relational ethics, I would like to propose a transdisciplinary solution that enables experimental, subject-specific, and practice-based research methods to enter into complementary, overlapping collaboration. I propose this moderate approach with gratitude for the critique raised in the more radical calls. This collaboration I suggest would include developing ethics protocols drawing on each of these fields' strengths in, respectively, procedural ethics, EDIA principles, and relational ethics.

As previously mentioned, a transdisciplinary framework enables inquiries to be pursued in multiple overlapping studies, each led and defined by the methods of an individual discipline. At the same time, these studies are informed by the complementary priorities of the others, and governed by any shared principles, guidelines, or frameworks adopted. In a performing arts psychology project, one study may be deeply anchored in the community's socio-behavioral engagement with a practice through participatory methods and relational ethics; another may be informed by critical theory and produce historically and culturally contextualized interpretations of practice with equity in mind; a third may work experimentally and produce generalized insights into cognitive demands and effects of the practice within a predefined ethics procedure. Across the three overlapping studies the psychology of a performance practice can be understood more holistically, through its socio-behavioral, contextual, historical, and cognitive aspects. This holistic understanding will also inform the selection of shared samples and sites. More importantly, however, the limitations of each method and ethical approach become more visible through the others at the early stage of designing the studies. It may help reveal limitations and opportunities to consider ethics across the triad, situate practice conceptions, and diversify beyond WEIRD paradigms.

This solution requires open negotiation of principles of methodological validity with willingness to adapt some norms and accommodate others. The aim is to ensure that every study can produce knowledge that is recognizable to its target group while establishing useful overlap between the studies. Indeed, I would like to suggest that the challenge of negotiating across methodologies in transdisciplinary research can be approached as a strength. It can motivate collaborators to seek the complementary ethical and methodological connections and compromises needed to address the WEIRD problem and the problem of overgeneralized practice concepts.

## Author contributions

PH: Writing – original draft.

## Footnotes

^1^It would be relevant to examine whether a (w)EIR(d) version of the sample problem exists outside of Western, democratic countries.

^2^This aspect of the WEIRD problem likely affects research practices beyond Western institutions because publishers and peer reviewers predominantly expect authors in/from the Global South to adhere to Western methodologies, which may inhibit the use of other knowledge systems, including Indigenous ways of knowing.

^3^For perspectives on what subject-specific fields may gain in turn (see Ross, 2018; Murphy, 2023).

^4^In performing arts disciplines, practice-based research (PBR), Practice as Research (PaR), artistic research, and similar approaches emerged in the late 1990s and early 2000s in response to the integration of conservatory programs into university institutions in and beyond Europe. Today, practice-based performing arts research journals, book series, conferences, working groups, and PhD degrees exist across the world.

^5^The term abduction refers to the third form of inference identified in Charles Sanders' Piece's theory of knowledge. Less known than bottom-up induction and top-down deduction, abduction allows novel, associative leaps to plausible, but more loosely empirically founded or logically tested, conclusions that then are brought back into inductive and deductive studies for verification.
